# Upper Extremity Examination for Neuromuscular Diseases (U-EXTEND): Protocol for a Multimodal Feasibility Study

**DOI:** 10.2196/40856

**Published:** 2022-10-27

**Authors:** Robert Gutierrez, Allison McCrady, Chelsea Masterson, Sarah Tolman, Mehdi Boukhechba, Laura Barnes, Silvia Blemker, Rebecca Scharf

**Affiliations:** 1 School of Engineering & Applied Science University of Virginia Charlottesville, VA United States; 2 Department of Biomedical Engineering University of Virginia Charlottesville, VA United States; 3 University of Virginia Children's Hospital Charlottesville, VA United States

**Keywords:** mHealth, ubiquitous computing, neuromuscular disorders, inertial measurement unit, motor function, specific torque, cross-sectional area, echogenicity

## Abstract

**Background:**

Neuromuscular diseases, such as spinal muscular atrophy (SMA) and Duchenne muscular dystrophy (DMD), may result in the loss of motor movements, respiratory failure, and early mortality in young children and in adulthood. With novel treatments now available, new evaluation methods are needed to assess progress that is not currently captured in existing motor scale tests.

**Objective:**

With our feasibility study, our interdisciplinary team of investigators aims to develop a novel, multimodal paradigm of measuring motor function in children with neuromuscular diseases that will revolutionize the way that clinical trial end points are measured, thereby accelerating the pipeline of new treatments for childhood neuromuscular diseases. Through the Upper Extremity Examination for Neuromuscular Diseases (U-EXTEND) study, we hypothesize that the novel objective measures of upper extremity muscle structure and function proposed herein will be able to capture small changes and differences in function that cannot be measured with current clinical metrics.

**Methods:**

U-EXTEND introduces a novel paradigm in which concrete, quantitative measures are used to assess motor function in patients with SMA and DMD. Aim 1 will focus on the use of ultrasound techniques to study muscle size, quality, and function, specifically isolating the biceps and pronator muscles of the upper extremities for follow-ups over time. To achieve this, clinical investigators will extract a set of measurements related to muscle structure, quality, and function by using ultrasound imaging and handheld dynamometry. Aim 2 will focus on leveraging wearable wireless sensor technology to capture motion data as participants perform activities of daily living. Measurement data will be examined and compared to those from a healthy cohort, and a motor function score will be calculated.

**Results:**

Data collection for both aims began in January 2021. As of July 2022, we have enrolled 44 participants (9 with SMA, 20 with DMD, and 15 healthy participants). We expect the initial results to be published in summer 2022.

**Conclusions:**

We hypothesize that by applying the described tools and techniques for measuring muscle structure and upper extremity function, we will have created a system for the precise quantification of changes in motor function among patients with neuromuscular diseases. Our study will allow us to track the minimal clinically important difference over time to assess progress in novel treatments. By comparing the muscle scores and functional scores over multiple visits, we will be able to detect small changes in both the ability of the participants to perform the functional tasks and their intrinsic muscle properties.

**International Registered Report Identifier (IRRID):**

DERR1-10.2196/40856

## Introduction

Spinal muscular atrophy (SMA) is a neuromuscular disease that occurs in approximately 1 in 11,000 live births [1,2]. SMA is characterized by severe muscle atrophy and weakness that are attributed to motor neuron degeneration. The cause of SMA is a variation of the survival motor neuron 1 gene that results in the death of the motor nerves, leading to reduced muscle size and function and increased muscle fibrosis [3]. Together, these changes cause severe muscle weakness and the atrophy of muscle, as observed in patients. The recent development of novel therapies, such as nusinersen [4], risdiplam, and onasemnogene abeparvovec (Zolgensma), which target motor neuron survival, has allowed for extended life expectancy and improved motor function. However, the previous lack of treatments for SMA and the coinciding lack of progress for children with SMA have resulted in a dearth of meaningful evaluation methods for precisely and quantitatively measuring muscle and motor function in these children to capture what is the most meaningful to them and their families.

Duchenne muscular dystrophy (DMD) is another progressive neuromuscular disease of childhood that occurs in 1 in 6000 boys [5]. DMD is caused by a lack of the dystrophin protein at the subcellular level in skeletal muscle. This missing linkage to the extracellular matrix results in a chronic state of inflammation and dysregulated muscle repair, leading to muscles being replaced with muscles with fibrosis and fat infiltration. This decrease of contractile muscle material results in the loss of motor movements, respiratory failure, and early mortality in young adulthood. New treatments are also being developed for DMD, and evaluation methods are direly needed.

The explosion of new potential treatments for these diseases presents the urgent need for more specific, more quantitative metrics that can be used to quickly and effectively evaluate motor function and, potentially, the efficacy of a treatment or the progression of these diseases. This is especially important for pediatrics because the standard metrics that are used for adults do not always apply to children and vice versa. Additionally, clinical trials are not inclusive of patients with cognitive challenges due to current analyses of clinical outcomes requiring patient participation. We recognized a critical absence of tools and systems for quantifiably and objectively capturing meaningful motor function in an inclusive way for this population.

By following these patients, we have identified multiple ways in which motor function evaluations could be dramatically improved. The Upper Limb Module (ULM) was developed as an international effort among clinicians, physical therapists, and researchers to address the shortcomings of previous SMA motor function assessments [6]. The ULM was targeted toward younger children and overcame the flooring effects found in the Hammersmith Functional Motor Scale-Expanded (HFMSE). However, the ULM had issues with the ceiling effect, which led to the creation of the Revised ULM (RULM) [6]. A few of the tasks that were added in the RULM included picking up coins, pushing a button light with 1 hand, and raising a 200-g cup to the mouth. Although the tasks were related to activities of daily living and allowed for a complete range of motion in different joints, the RULM does not explicitly measure each task. The HFMSE and Children’s Hospital of Philadelphia: Infant Test of Neuromuscular Disorders [7] are neurological assessments for infants; however, patients in our clinic currently range in age from 0 (birth) to 35 years. Therefore, the metrics of these assessments are less relevant to many of our patients. Further, these assessments are performed in clinics and therefore may have less relevance to a patient’s functions in their normal daily environments. This has been frequently observed by parents and caregivers, who have reported progress that is not captured by standard clinical assessments. Finally, the current assessments are qualitative and may not be able to capture specific changes in muscle strength and function.

Ultrasound imaging may be used to measure architectural characteristics of skeletal muscle. These architecture measurements can be used to estimate the force-generating capacity of a muscle based on the established relationships between muscle size and function [8]. Recently, ultrasound imaging has been used to measure disease progress directly in vivo for various neuromuscular disorders, including SMA and DMD, by measuring tissue echogenicity (brightness) or increased fibrosis [9-14]. Fibrosis leads to a loss of contractile muscle tissue, thereby reducing the effective force-generating capacity of the muscle. It has been observed that patients with SMA exhibit severe muscle atrophy and increased fibrosis in their muscles [13]. However, there is no standard quantification method for either of these characteristics. Increasing the understanding of the structural changes in SMA and DMD will help us to better understand how the force-generating capacity decreases throughout these diseases’ progression.

As wearable technology becomes more ubiquitous, mobile health apps have grown immensely. Wearable sensors are often embedded in smartphones, smartwatches, and many app-specific devices. Previous efforts have attempted to score motion assessments via various modalities of motion tracking technology, from cameras to inertial measurement units [15,16]. Many of these works used cameras and multiple wireless sensors to track upper limb movement to report angles of motion [17,18]. However, these studies focused on classifying movements according to the assessment scale rather than quantifying movements directly, which can introduce experimenters’ biases into the algorithm.

To more precisely track the changes in muscles and functions among patients with SMA, DMD, or other neuromotor disorders, we must first understand what we can easily measure in a clinic setting. By improving function tracking in clinics via methods that are more objective and sensitive, we will be able to better recognize functional changes that are indicative of treatment efficacy. We will combine measurements of tissue quality and muscle size to estimate muscle function based on image-based methods. We will use wireless sensors to quantify the quality of motions and make more nuanced comparisons than those that can be performed with nominal clinical assessment ratings. By combining imaging methods that provide theoretical estimates of function and wireless sensor measurements of actual function, we can better understand the changes that occur in muscle structure and how they result in functional changes that are reflected by sensor measurements. These proposed novel methods for assessing muscle and motor function will be easily implemented into the standard of care and normal clinical visits. In summary, our proposed research is novel, and it will advance the treatment of patients with neuromuscular disease.

We propose a research program to develop sensitive and novel measures for muscle function in patients with SMA and DMD. We have found these measures to be greatly needed in our clinical care, as well as in the research that is so vital to promoting treatments for these patients. Our pilot study introduces a novel paradigm in which concrete, quantitative measures are used to assess motor function in patients with SMA and DMD. The major developments include leveraging (1) ultrasound imaging and handheld dynamometry to measure and comparatively track upper extremity muscle structure and function over time and (2) motion mechanics measurements by using accelerometers and gyroscopes with wireless sensing technology.

## Methods

### Study Design

In collaboration with the University of Virginia Children’s Hospital Pediatric Neuromuscular Disease Clinic, which is run by RS, we will recruit a cohort of patients with SMA (n=9) and patients with DMD (n=20) and a cohort of age- and sex-matched healthy controls (n=15). For the age matching process, control participants will be accepted if they are aged within 1 year above or 1 year below the patient participants, allowing 1 control participant to serve as the control for multiple patient participants in the study. These participants will be recruited and consented according to the institutional review board–approved procedures. Participants will range in age from 0 (birth) to 35 (adulthood) years. At routine checkups (generally conducted every 4-6 months), we will measure upper extremity muscle size, quality, function, and contraction strength. We will examine the data for distributions and look at trends in muscle measurements across the study cohorts and over time. We will generate scores for theoretical muscle function and the quality of motor movements. This information will be used to compare a patient's muscle structure and improvements over time.

We performed initial pilot studies to determine the feasibility of ultrasound imaging and the relationship between the ultrasound imaging data and the wearable sensor data. Our exploratory study was designed to recruit as many patients from the clinic as possible to determine the characteristics of muscle structure and motor function in each neuromuscular disease. We plan to develop an image-based theoretical measure of muscle function and a movement function score; correlate the muscle function measures to the movement function scores; and then aggregate them into a functional score, which we will correlate to the clinical function assessment scores.

### Aim 1: Ultrasound Measurements of Muscle Architecture

Ultrasound images will be collected to measure biceps brachii and brachioradialis muscle structures in participants with SMA and DMD and age- and sex-matched healthy controls. A linear array transducer (H9.0/40, LS 128 CEXT; Telemed Medical Systems) will be used, with settings kept constant across participants to allow for consistent measurements. The depth will be kept at 50 mm, the frequency will be kept at 8 Hz, and the gain will be set to 88%. The dominant arm’s biceps brachii and brachioradialis muscles will be imaged at the approximate midsection in the longitudinal and transverse planes. The maximum voluntary elbow flexor torque will be measured by using a handheld dynamometer (Chatillon DFS II; John Chatillon & Sons Company), which will be placed on the forearm at a measured distance from the elbow joint center, with the elbow positioned at approximately 90° (flexed). The measured distance will then be multiplied by the force measurement from the dynamometer to calculate elbow torque. We will collect 5 measurements and average them to calculate the maximum voluntary elbow flexion torque.

**Figure 1:**
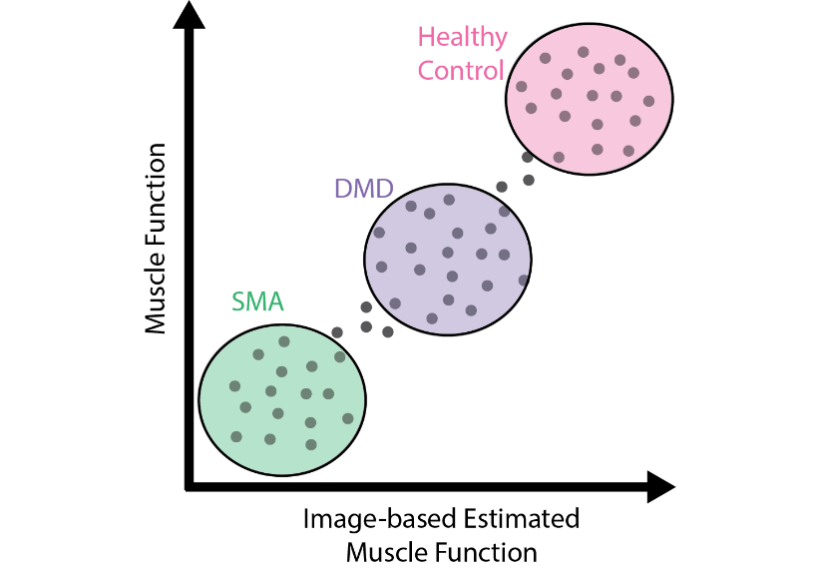
A theoretical comparison between image-based estimates of muscle function and measurements of muscle function. DMD: Duchenne muscular dystrophy; SMA: spinal muscular atrophy.

A custom image processing algorithm that was developed in MATLAB (The MathWorks Inc) [19] will be used to measure the cross-sectional areas (CSAs) and average echogenicities of the muscles. In total, 5 images of the same region will be measured and averaged to generate 1 average measurement for a participant. The CSAs will be manually traced for each transverse image frame, and the areas will be computed by calculating the number of pixels in the region of interest and converting this measurement to squared centimeters. To compare CSAs across cohorts, CSAs will be normalized by forearm length to mitigate the effects of participants’ size. The average echogenicity of the transverse plane will be calculated from the manually traced areas by averaging the pixel grayscale intensity values, which range from 0 (black) to 256 (white). The average echogenicity of the longitudinal plane will be calculated by manually drawing a rectangle across the region of interest in the muscle and averaging the pixel grayscale intensity values. The two planar echogenicities will then be averaged to create 1 measurement of echogenicity for each muscle and to minimize transducer orientation effects. A measure of muscle function or the amount of torque per unit area (specific torque) will be estimated by dividing the elbow flexor torque (N·m) by the CSA (cm^2^). We will then generate an image-based estimate of muscle function by using the abovementioned calculated parameters. We will validate this metric by comparing it to the elbow flexor torque, which will be a uniform measure of muscle function across all participants (Figure 1). All statistics will be calculated in R (R Foundation for Statistical Computing; α=.05) [20].

### Aim 2: Inertial Measurement Unit Measurements of Arm Function

**Figure 2:**
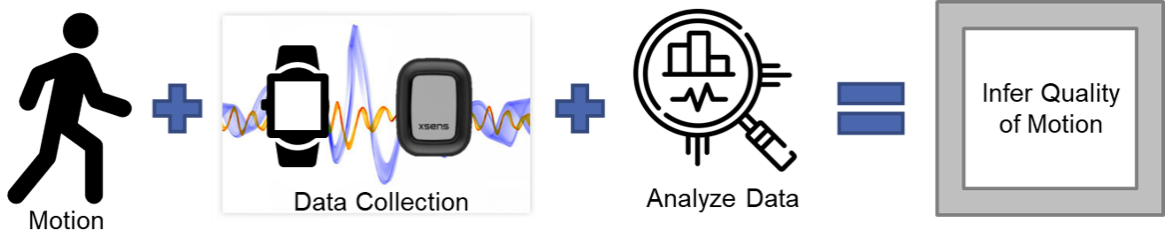
Upper Extremity Examination for Neuromuscular Diseases methodology overview. Participants will perform a set of motions that will be measured via ultrasound imaging and wearable sensors. The data will then be analyzed to infer the quality of motion metrics.

**Figure 3:**
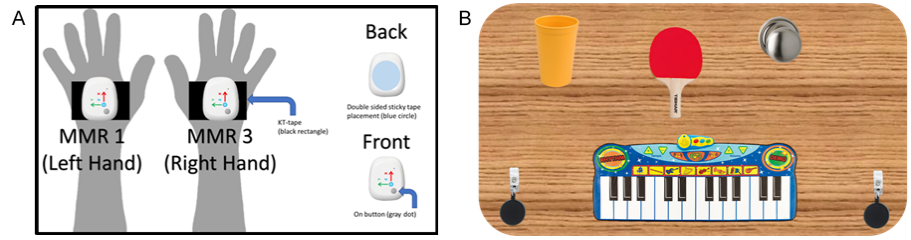
Wearable sensor diagram (A) and activity board prototype (B). KT Tape: Kinesiology Therapeutic Tape; MMR: MetaMotionR+.

We will use multimodal wireless inertial sensors (MetaMotionR+; Mbient Lab) and the Microsoft Kinect V2 camera for motion tracking to measure movements in space (Figure 2). Attaching sensor nodes to a participant’s hand (Figure 3) will allow us to measure progress with medication or during physical therapy in small gradations to detect micromovements. We will gather measurements from dominant and nondominant arms by having the child participants press against the manometer and pinch the grip strength gauge with each hand or arm. Through a series of prescribed motions (similar to those in the RULM for SMA; eg, picking up tokens and raising a cup), participants will perform tasks that are related to everyday activities, with the help of the activity board (Figure 3).

The list of tasks includes turning a door knob; performing a closed-fist knock; performing paddle pronation and supination; performing the finger-thumb test; opening and closing the fist; pressing a piano key; raising a cup to the mouth; performing a bicep curl; performing a hand clap; and rotating the shoulder sideways, forward, and backward. For each task, 3 measurements will be collected, and the strongest measurement for each task will be used. The wireless sensing technology will be first piloted with participants from the healthy control group to adjust and calibrate our protocol. The technology will then be used with the full cohort of 20 patients. These controlled data collection processes will be done in the clinic to drive the development and validation of the sensing requirements (ie, for both the sensors and the sampling rates) and data analytics methods for conducting the motion assessments with our patient population, thereby allowing for subsequent out-of-clinic deployments for continuous monitoring.

### Ethics Approval

The study was approved by the University of Virginia’s Institutional Review Board for Health Sciences Research (protocol number: 200178) on September 25, 2020. The participants or the parents of the minor participants provided written informed consent for participating in the study.

## Results

Data collection for both aims began in January 2021. As of July 2022, we have enrolled 44 participants (9 with SMA, 20 with DMD, and 15 healthy participants). We expect the initial results to be published in summer 2022.

## Discussion

### Study Overview

We hypothesize that by applying the described tools and techniques for measuring muscle structure and upper extremity function, we will have created a system for the precise quantification of changes in motor function among patients with neuromuscular diseases. Since traditional motor function assessments provide scores on a scale, patient assessment scores remain relatively the same, despite patient-reported improvements. Our study will allow us to track the minimal clinically important difference over time to assess progress in novel treatments. From a clinical standpoint, a physician could track a patient’s movements in reference to the control population, as well as in reference to a patient's initial baseline. Therefore, regardless of the amount of movement, every patient will receive a quantitative score for the movements that they can do, which can be tracked for future comparisons. Similarly, the scores and the overall motion shapes can be used to understand range of motion limitations, fatigue, and the impact of novel therapies.

The ability to use ultrasound imaging to assess muscle architecture will not only lower the financial burdens of patients, insurance companies, and hospitals but also reduce the stress of patients. We expect that our measures will allow us to identify early changes in muscle architecture and may ultimately be able to predict long-term improvements or deteriorations in function. By comparing the muscle scores and functional scores over multiple visits, we will be able to detect small changes in both the ability of the participants to perform the functional tasks and their intrinsic muscle properties.

The potential impacts of our pilot study are revolutionizing the way that clinical trials are performed and accelerating the pipeline of new treatments for childhood neuromuscular diseases. This protocol is especially important for pediatrics because the standard metrics that are used for adults do not always apply to children. By tracking incremental changes, our study will also aid in the development of a methodology for identifying which treatments support the progression or sustainment of muscle structure and function. The proposed assessment could drive the development of software and applications that aid occupational and physical therapy, and it could provide a more objective, quantitative way of evaluating functional upper extremity motions and the quality of such movements. Our study will also help pave the way for home-based activity tracking, which can ease the burdens of patients and their families. Our results will have the potential to inform treatment efficacy and help provide evidence for making decisions regarding the effectiveness of SMA and DMD therapies.

### Dissemination Plan

The results from our research will be presented at relevant technical and clinical academic conferences and published in scholarly peer-reviewed publications. As we are a highly interdisciplinary team, per our dissemination plan, we will aim to reach diverse audiences within the systems engineering, biomedical engineering, and medicine fields. We also anticipate sharing our findings with care teams for patients with neuromuscular diseases.

### Limitations

The primary limitation of our study is the large variations in motor function between the healthy controls and the patients. Comparing control functionality to patient functionality has proven to be difficult, since patient functionality can be vastly different; however, we are focused on capturing more precise measures at the lower end of the functional scale and are not very concerned about any potential ceiling effects at the higher end of the functional scale. Patient participation can be affected by neurodevelopmental challenges, such as autism and intellectual disabilities, resulting in partial or inadequate data collection. This limitation could be mitigated through the inclusion of additional supports to allow for improved participation. External factors, such as medical illnesses or additional medical appointments, may also limit attendance for data collection.

### Conclusion

Our study is poised to make a valuable contribution to the understanding of functional changes and advance the care of the patients. We see particularly exciting opportunities for translating this work into the clinic to support current clinical assessments by providing additional information on detailed changes in muscle function. Our next steps include (1) expanding the number of participants that are longitudinally tracked to further validate our novel functional metrics, (2) including additional raters (clinicians, radiographers, physical therapists, and occupational therapists), (3) expanding to other sites to explore our metrics’ wider applicability to other clinic settings; and (4) applying our framework to other neuromuscular diseases to provide detailed assessments of motor function to other patient groups.

## Appendix

Multimedia Appendix 1 Peer-review report by the University of Virginia - Center for Engineering in Medicine - Engineering-in-Medicine Seed Grant Program (Virginia, USA).

## Abbreviations

CSA: cross-sectional area
DMD: Duchenne muscular dystrophy
HFMSE: Hammersmith Functional Motor Scale-Expanded
RULM: Revised Upper Limb Module
SMA: spinal muscular atrophy
U-EXTEND: Upper Extremity Examination for Neuromuscular Diseases
ULM: Upper Limb Module

## Data Availability

The data sets that will be generated and/or analyzed during the study will not be publicly available due to patient confidentiality and data sharing agreements. Data may be available from the corresponding author on a case-by-case basis.
