# Supplementary material for: Upper Extremity Examination for Neuromuscular Diseases (U-EXTEND): Protocol for a Multimodal Feasibility Study
Source: JMIR Res Protoc. 2022 Oct 27;11(10):e40856. doi: 10.2196/40856 (PMC9650577; doi:10.2196/40856)
Supplement: Multimedia Appendix 1 [file resprot_v11i10e40856_app1.pdf]

ENGINEERING-IN-MEDICINE SEED GRANT PROGRAM
Review Summary

Project Title
Upper EXTremity Examination for Neuromuscular Diseases (U-EXTEND):
Technology-enabled Methods to Assess Treatment Efficacy and Accelerate Drug Discovery

Co-PIs
Barnes (SEAS – ESE); Scharf (SOM – Pediatrics); Blemker (SEAS – BME); Boukhechba (SEAS – ESE)

Ratings:

Fostering Collaboration:	**Reviewer 1:** Top 25%	**Reviewer 2:** Top 10%
Lead to External Funding:	**Reviewer 1:** Top 10%	**Reviewer 2:** Top 25%
Scientific/Clinical Impact:	**Reviewer 1:** Top 10%	**Reviewer 2:** Top 10%
Overall:	**Reviewer 1:** Fund	**Reviewer 2:** Fund

EIM Feedback to co-PIs
This revised proposal brings together a pediatrician (Scharf), a biomedical engineer who studies muscle mechanics (Blemker), a systems engineer with experience tracking motion using wireless sensors (Barnes), and a computer scientist with mobile health and clinical monitoring experience (Boukhechba). Together, they seek to develop better methods for monitoring changes in upper extremity motor function in children with spinal muscular atrophy (SMA). In response to previous reviews, the team has done a nice job of refining their study design and methodology; they also have an excellent embedding plan.

Reviewer Feedback to co-PIs

Reviewer 1
Thank you for resubmitting this grant. The objectiveness of the tool you are attempting to develop could be a game changer. Having the three groups (including a control) and changing to a cross sectional study is definitely an improvement. In addition, it appears the US technology for objective measures is more refined than previous application. In the manual processing the researchers may want to perform an inter-rater reliability assessment for persons manually processing images. Your embedding plan is robust and it appears that cross pollination between the medical and engineering groups is already occurring. The timeline is compressed to seven months, unsure why when previous application was 0,4,8 months data collection. Follow-on funding targets seem appropriate.

Reviewer 2
This proposal brings together a pediatrician (Scharf), a biomedical engineer who studies muscle mechanics (Blemker), a systems engineer with experience tracking motion using wireless sensors (Barnes), and a computer scientist with mobile health and clinical monitoring experience (Boukhechba). Together, they seek to develop better methods for monitoring changes in upper extremity motor function in children with spinal muscular atrophy (SMA). The application makes a good case that there is a need for better methods to track structural and functional changes in children with neuromuscular disease, and it appears highly likely that this team could develop such methods. In this revised application, the team presents compelling plans for exchange of personnel (embedding) between the clinical and engineering environments, for testing their measurement methods across 3 clinical groups with a wide range of function (SMA, DMD, normal), and for differentiating their work from other studies in the field by focusing on upper extremity function. Overall this is an excellent revised proposal.
